# Esophageal Foreign Body: A Case Report of a Refractory Croup in a 20-Month-Old Boy

**Published:** 2016-11

**Authors:** Sevil Nasirmohtaram, Nooshin Shabani

**Affiliations:** 1 *Otorhinolaryngology Research Centre, Gilan University of Medical Sciences, Amir-almomenin Hospital, Rasht, Iran.*

**Keywords:** Barky cough, Croup, Esophageal foreign body, loquat

## Abstract

**Introduction:**

Foreign body ingestion is common among children and more common in boys and in children under the age of 3. It can present with a wide variety of symptoms like dysphagia and drooling or symptoms related to the upper aerodigestive tract.

**Case Report:**

A 20-month-old male presented with refractory croup and poor feeding since 2 weeks. Bronchoscopy and esophagoscopy was performed due to suspicious history of eating loquat. The core of the fruit was found in the esophagus.

**Conclusion:**

Physicians should be aware of the variability of esophageal foreign body presentations to prevent serious complications due to delay in diagnosis.

## Introduction

Foreign body ingestion is common and may cause morbidity and mortality in the pediatric population. Their diagnosis may be as simple as taking a history or may be challenging by observing vague respiratory symptoms, like life-threatening airway compromise, subtle respiratory symptoms or even no symptoms (1). A high level of clinical suspicion can prevent delays in diagnosis and subsequent complications and is also cost-beneficent to the health system. Esophageal foreign bodies are more frequent in boys and younger children. It can present with various symptoms like dysphagia‚ odynophagia, drooling, and sometimes with nonspecific respiratory symptoms (2).

## Case Report

We were consulted for a 20-month-old male child because of refractory croup. He was admitted in the pediatric hospital 2 days before the consultation and was treated as croup (ventoline, oxygen, and a single dose of steroid), which was unresponsive. In our first visit, his mother said the patient ate a loquat about 2 weeks ago and, during eating, had attack of belching and coughing. She claimed that she took out the core of the fruit from his mouth. However, her history was not very reliable due to their low socioeconomic status and due to the baby seeming to be neglected. After that the baby had suffered from a mild barky cough, which gradually progressed, and a loss of appetite

The child had a history of esophageal atresia, which was reconstructed neonatally. However, he did not have any problems with eating until 2 weeks ago. 

Upon physical examination, no drooling and respiratory distress was found but a severe barky cough was observable. During lung auscultation there was a bilateral coarse crackle and the patient was ill. On chest radiography, emphysema or atelectasia was not observed, but there was mild interstitial pattern.

**Fig 1:**
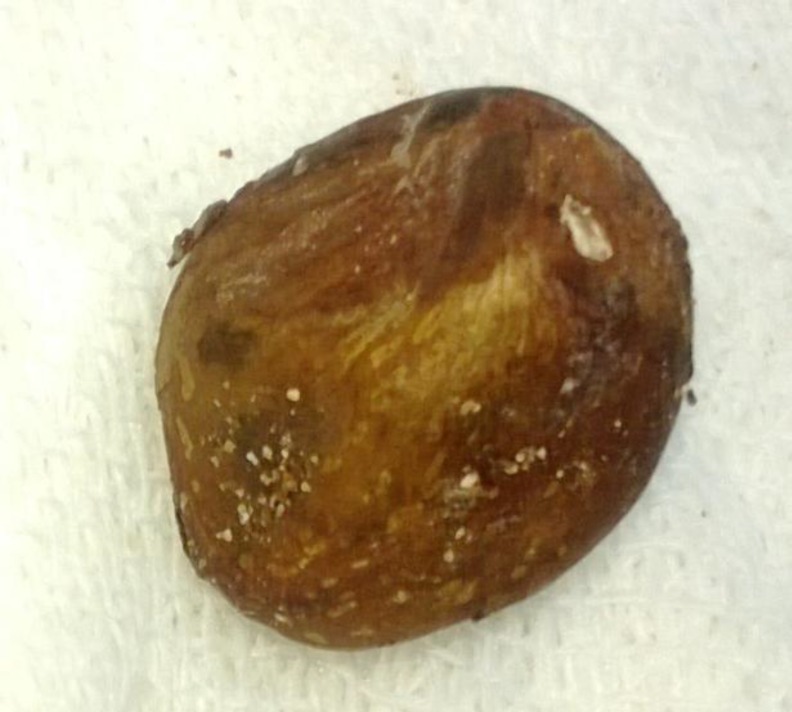
The core of loquat was found in rigid esophagoscopy

The patient was recommended for rigid bronchoscopy due to the suspicious history of eating a loquat. Upon bronchoscopy, no foreign body was found‚ but there was a compression on the posterior wall of the subglottis and purulent secretions in the trachea and both main bronchi. After‚ due to his past medical history, the patient underwent rigid esophagoscopy. In the esophagoscopy, we found a mass just below the cricopharynx. We used punch forceps and realized that there was an entire loquat core that had a compressive effect on the posterior wall of the subglottis and trachea (Fig.1). No stenosis was seen in the esophagus. After the foreign body was extracted, the signs of croup were relieved. He also received antibiotics for pneumonia and two days later significant changes in general condition was observed.

## Discussion

Esophageal foreign body can present with a wide variety of symptoms and is a possibility that should be considered. Respiratory symptoms are not necessarily a sign of a problem in the respiratory tract. Children may even be asymptomatic, so a high level of suspicion is needed especially in cases where ingestion of a foreign body was not witnessed. In some cases, history alone is enough to start further evaluation (3). Ingestions can vary in presentation from an asymptomatic state to respiratory distress or acute abdomen (4).

In 1991‚ Mileder P and colleagues reported an eleven-month-old infant girl who presented with a two-month history of inspiratory stridor. Barium swallow revealed an esophageal foreign body with tracheal compression. Upon endoscopy, a chestnut shell was extracted from the esophagus (5).

In 2003‚ Jacob Urkin and Yair Bar-David reported an 8-year-old boy who presented with a cough and mild respiratory difficulty and drooling. They found a marble ball just above the thoracic inlet, pushing the posterior wall of the trachea anteriorly and narrowing the lumen (3). In 2008‚ Kim N and colleagues reported a 7-week-old girl with a 3-week history of progressively worsening stridor. She was admitted to rule out a congenital anomaly in the airway. After work up, an esophageal foreign body causing esophagitis and proximal airway compression was found in imaging (6).

## Conclusion

Esophageal foreign body is a common problem, especially among children, which has the potential to cause significant and even life-threatening complications. Physicians should be aware of the variability of esophageal foreign body presentations and bear in mind the diagnosis in any child with symptoms related to the upper aerodigestive tract.
